# Preclinical toxicity evaluation of AAV for pain: evidence from human AAV studies and from the pharmacology of analgesic drugs

**DOI:** 10.1186/1744-8069-10-54

**Published:** 2014-09-02

**Authors:** Josef Pleticha, Lukas F Heilmann, Christopher H Evans, Aravind Asokan, Richard Jude Samulski, Andreas S Beutler

**Affiliations:** 1 Departments of Anesthesiology, Oncology, and the Cancer Center, Mayo Clinic, Rochester, MN, USA; 2 Rehabilitation Medicine Research Center, Mayo Clinic, Rochester, MN, USA; 3 Gene Therapy Center, University of North Carolina, Chapel Hill, NC, USA

**Keywords:** Adeno-associated virus, Pain, Gene therapy, Toxicology, Interleukin-10, Beta-endorphin

## Abstract

Gene therapy with adeno-associated virus (AAV) has advanced in the last few years from promising results in animal models to >100 clinical trials (reported or under way). While vector availability was a substantial hurdle a decade ago, innovative new production methods now routinely match the scale of AAV doses required for clinical testing. These advances may become relevant to translational research in the chronic pain field. AAV for pain targeting the peripheral nervous system was proven to be efficacious in rodent models several years ago, but has not yet been tested in humans. The present review addresses the steps needed for translation of AAV for pain from the bench to the bedside focusing on pre-clinical toxicology. We break the potential toxicities into three conceptual categories of risk: First, risks related to the delivery procedure used to administer the vector. Second, risks related to AAV biology, i.e., effects of the vector itself that may occur independently of the transgene. Third, risks related to the effects of the therapeutic transgene. To identify potential toxicities, we consulted the existing evidence from AAV gene therapy for other nervous system disorders (animal toxicology and human studies) and from the clinical pharmacology of conventional analgesic drugs. Thereby, we identified required preclinical studies and charted a hypothetical path towards a future phase I/II clinical trial in the oncology-palliative care setting.

## Introduction

Unrelieved chronic pain is a critical health problem in the US and worldwide. A report by the Institute of Medicine estimated that 116 million Americans suffer from pain that persists for weeks to years, with resulting annual costs exceeding $560 million [1]. Pain is an especially common problem in patients with advanced cancer, with some studies reporting that the majority of patients dying from metastatic solid tumors experience severe unrelieved pain despite treatment with available analgesics [2,3]. Antineoplastic treatment options have often been exhausted for these patients, placing palliation of symptoms at the center of treatment goals for the limited remaining life span, typically weeks to a few months. The combination of the great need for pain relief and of the presence of an underlying incurable disease creates scenarios for early clinical testing of the most novel (and potentially risky) new pain treatment strategies, first in selected patients with cancer pain as a phase I clinical trial. Therefore, the subsequent review on clinical translation and preclinical toxicology studies is predicated on a future plan to translate AAV gene therapy first in the palliative oncology setting.

Two vector systems have been most extensively studied in gene therapy for pain: Herpes simplex virus (HSV), which has already been tested in cancer pain patients [4]; and adeno-associated virus (AAV). AAV, the subject of this review, has not been tested clinically for pain. However, AAV is arguably the most advanced and widely studied vector in terms of clinical trials seeking long-term gene expression in non-malignant tissues [5]. The first AAV therapeutic product, alipogene tiparvovec, is now marketed in Europe [6]. Therefore, substantial pre-clinical toxicology data and clinical phase I/II toxicology research exists for AAV that appears applicable to the translational development of this vector in the field of pain.

## Defining the scope of possible toxicities

**Figure 1:**
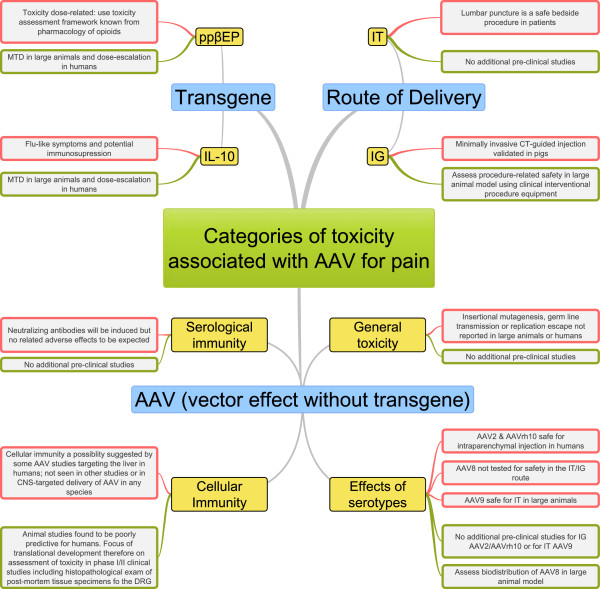
**Potential toxicities of adeno-associated virus (AAV) gene therapy for chronic pain.** Risks were sorted into three mutually exclusive categories (blue). For each type of risk existing evidence is highlighted (red) and future studies towards pre-clinical toxicology are proposed (green). MTD, maximum tolerated dose; CNS, central nervous system; IG, intraganglionic; IT, intrathecal; DRG, dorsal root ganglia; ppßE, prepro-ß-endorphin; IL-10, interleukin-10.

The potential toxicities of AAV for pain can be conceptually broken down into mutually exclusive categories of risk. This review divides toxicities as follows: the procedure employed for vector delivery to the target tissue, i.e., a surgical/interventional risks (section 3); the AAV vector itself (*sin* transgene), i.e., viral and immune-biology (section 4); and the specific therapeutic gene, i.e., pharmacology (section 5). An overview is provided as a semantic “mind map” in Figure 1.

## Potential toxicities related to the procedure employed for vector delivery

Two routes of delivery have been used in rodent models to demonstrate analgesic efficacy of AAV through gene transfer to the nociceptive neurons in the DRG: intrathecal (IT) or intraganglionic (IG) administration.

The IT route delivers the vector to the cerebrospinal fluid (CSF) and has been achieved in rats by inserting an IT catheter through the *cisterna magna* and advancing it caudally to the lumbar level [7-10]. The same approach has been used in dogs [11,12]. In pigs, we recently reported an alternative: direct injection into the lumbar CSF by a lateral lumbar puncture under interventional image guidance with a computed tomography (CT) scanner [13]. In humans, IT delivery can be easily performed by lumbar puncture (LP), a routine bedside procedure with excellent safety profile [14].

Therefore, no additional toxicity studies would be needed in regards to the LP procedure risk, if a phase I trial of AAV for pain were to test vector administration by the IT route.

The IG route delivers AAV directly into the DRG parenchyma. IG studies in rats accessed the DRG by an open neurosurgical procedure [15-19], which would not be attractive in humans because it would require a complicated and invasive procedure. We therefore developed a minimally invasive, CT imaging-guided technique to safely target the DRG in the pig model. Using a customized needle assemble for convection enhanced delivery (CED), we then demonstrated efficacy of CED to deliver AAV into the DRG parenchyma [20]. Comparative anatomy analysis of the human and the porcine spine suggested that the technique will be easily translatable to humans. As a matter of fact, the imaging equipment used in our study was identical with the state-of-the art equipment currently used for neuroradiological spine interventions in humans.

In the field of interventional neuroradiology, it is common practice to target small structures such as the DRG for drug injections or biopsy. Ultrasound ablation of the spinal sensory nerve root has also been reported [21]. Therefore, while IG drug delivery is a new clinical procedure, it will resemble clinical practice of similar interventions. Accordingly, we conclude that only a limited scope of additional preclinical toxicity assessment of the IG injection procedure might be needed, e.g., to determine absence of DRG tissue damage, and that such studies can be performed in the pig model of the procedure that we have already in place.

## Potential toxicities associated with the AAV vector: Viral- and immune biology

Clinical translation of AAV for pain faces the issue of general vector toxicity: the risk of AAV itself (without the transgene, which will be discussed in the Section 4 below). This section is predicated on the conservative assumption that vector may spread systemically to all organ sites, even though the IT and IG route of administration attempts to limit such spread.

Previous experience with AAV gene therapy identified several mechanisms by which the vectors trigger an adverse reaction; each is addressed in a separate subsection below.

### Vector related general toxicities: insertional mutagenesis, germ line transmission, and replication escape

AAV vectors persist in cells in two states, as episomal concatamers and, for a small minority of vector copies, chromosomal integration [22]. In a specific experimental setting of newborn mice, AAV vectors were found to integrate into the genomes of hepatocytes at a rate of 0.1 – 1% of transduction events [23,24] resulting in the development of hepatocellular carcinoma [25]. While this report generated substantial concern in the field, subsequent investigation by numerous laboratories have suggested that the observation is likely not applicable to AAV gene therapy in adult humans. No evidence of insertional mutagenesis was reported after systemic delivery of AAV in humans or large animals models or after selective delivery to the CNS in any species.

Transmission to the germ line has not been found in any AAV clinical trial. While AAV particles were detected in the semen of subjects receiving AAV2 into the hepatic artery [26], this was temporary.

The potential of replication escape of the recombinant AAV was shown *in vitro* in the setting of co-infection with the wild type AAV2 and a helper virus [27]. However, it has not been reported in any of the AAV preclinical toxicology studies or subsequent clinical trials.

The most serious adverse event in the setting of a AAV human trial occurred in 2007 when a patient died 22 days after receiving intra-articular AAV encoding etanercept, a tumor necrosis factor receptor blocker [28,29], from a large retroperitoneal hematoma and systemic histoplasmosis. The FDA stopped the clinical trial but, after an investigation, allowed the trial to resume. Similar adverse events were not seen in any of the other patients.

In regards to AAV for pain, the above evidence suggests that issues of insertional mutagenesis, germ line transmission, replication escape, and dissemination of the vector to the public domain, have been so thoroughly addressed in the field. The adverse event in the AAV-etanercept trial suggests that in the case of IL-10, a transgene that is similar to etanercept in regards to its immune-suppressive effect, measurement of systemic levels should be an important goal of preclinical toxicity assessments. Furthermore, a pain trial in patient with incurable cancer could answer some of the above questions in humans, at least if (some of the) patients may decide that after their (at trial entry anticipated) death from cancer (i.e., at the end of the trial) autopsy tissue may be sampled for research purposes.

### Immune response to the AAV vector (sin transgene)

AAV for pain by the IT or IG route will trigger a serological immune response to the vector capsid. In addition, cellular immunity to the vector capsid has to be carefully considered, although (or because) fewer predictions may be possible in regards to the cellular immunity one may anticipate in humans.

#### Serological immunity

AAV delivered IT or by direct infusion into the brain parenchyma led to >100-fold increase in the titer of circulating NAb in large animal models as shown for AAV2 [30,31], AAV1 [32], AAVrh10 [33], and AAV9 [34,35]. The measured titers of circulating NAb seemed to be proportionate to the total dose of AAV vector administered. The clinical trials employing intraparenchymal infusion of AAV to the brain also demonstrated a rise of circulating NAb in some but not all patients [36-42]. NAb titers in the CSF were examined only in some of these studies. When measured, the CSF NAb levels were consistently lower compared to the systemic NAb, suggesting the systemic origin of the humoral immune response. Importantly, while NAb occur, they have no pathological consequence: No clinical symptoms or adverse effects were associated with the presence of NAb in any animal or human study.

The presence of pre-existing circulating NAb inhibits AAV transduction when the vector is delivered systemically [43]. In regards to IT AAV, studies in large animal models suggest a threshold effect for the capacity of systemic NAb to prevent AAV transduction. In non-human NHP, transduction by IT AAV9 was inhibited by NAb titer of ≥ 1:200 but not affected by titers of ≤ 1:128 [35,44]. Studies in dogs performed by Haurigot et al. [34] demonstrated only moderately reduced CNS- and PNS transduction rates for NAb titers as high as 1:1000, suggesting that an immune privilege-like status may apply to the PNS. Pre-existing systemic NAb did not affect intraparenchymal gene transfer to the brain by any serotype in either NHP [31] or humans [41].

For the development of AAV for pain, we conclude that the vector will induce NAb without any pathological consequences. Pre-existing NAb may diminish IT transduction rates, but the extent or threshold is a clinical research question (of minor importance) that may be answered in a clinical trial. Pre-existing NAb will not affect IG delivery of AAV. Taken together, we propose that a phase I trial of AAV for pain should collect specimen to measure NAb before and after vector administration, while no further preclinical studies are required towards that plan. Preexisting NAb should not be an exclusion criterion for individual patients considered for IT or IG AAV for cancer pain.

#### Cellular immunity

The clinical significance of CD8 mediated cytotoxicity directed against the AAV capsid and leading to a destruction of the transduced cells arose as an issue in a clinical trial of AAV2 for hemophilia B [26]. Importantly, the precedent preclinical studies had failed to predict the cellular immune response in animal models [45].

Subsequent animal and human studies demonstrated that the extent of cellular immune response against AAV might depend on the host species, the AAV serotype, and the target tissue. A comparison of rodents, large animal species, and humans showed that humans are prone to mount a cellular immune response to systemic AAV more readily than other species [46,47]. The choice of AAV serotype may affect the cellular immunity through direct, antigen presenting cell-independent activation of T-lymphocytes by an AAV capsid [48,49]. When compared to systemic administration, delivery of AAV directly to the nervous system did not result in cell-mediated immunity to the AAV capsid for either the intraparenchymal route, tested in both large animals and humans [32-34,36,41,42,50-56] or the IT route, tested to date in large animals only [34,57,58].

Based on this evidence, the occurrence of a cellular immune response against the AAV capsid in the PNS cannot be definitely excluded on the basis of large animal studies. It will therefore be a critical focus of a phase I clinical trial of AAV for pain, which will need to include collection of peripheral mononuclear blood cells and extensive *post-mortem* histopathological analysis for possible immune-toxicity. This approach appears particularly justified by the absence of cellular immunity-associated adverse effects in the context of previous AAV studies in the CNS. Furthermore, a cellular immune response could be effectively managed by immunosuppression [36,49], suggesting that this option, i.e., use of systemic corticosteroids, should be allowed in a clinical trial of AAV for pain if immune toxicity, e.g., a Guillain-Barre like syndrome is seen in a patient. In addition, an AAV for pain human trial may provide unique opportunities for insights into the cellular immunity against AAV in the nervous system, if collection of post-mortem tissue can be accomplished.

### AAV serotypes and off-target biodistribution

In order to avoid toxicities and facilitate clinical translation of AAV for pain, selection of AAV serotypes will play a critical role. The ideal serotype for AAV for pain would effectively transduce PNS neurons after IT or IG administration; show low levels of escape to peripheral organ sites; and have precedent safety data available from a clinical trial (for another disease).

AAV8 effectively transduced the DRG neurons, while showing limited brain penetration upon IT administration in rodents [59-61], which might limit toxicities associated with CNS transduction. However, AAV8 manifests strong hepatotropism after systemic administration (and has thus been a leading contender in the preclinical and clinical studies of hemophilia B) [49]. While biodistribution of IT AAV8 has not been examined in a large animal model, any systemic spread of the vector would likely lead to transduction of the liver, raising the issue of systemic transgene effects.

IT AAV9 transduces both the PNS and CNS [34,35,44,62,63] and is in the final stages of preclinical development for mucopolysaccharidosis IIIA and giant axonal neuropathy [34,58], with studies suggesting an excellent safety profile for IT doses of up to 2 × 10^13^ genome copies (GC) in NHP (with projected dose of 3.5 × 10^13^ GC in humans). Widespread CNS transduction may be a characteristic of AAV9 and may appear less attractive for gene therapy for pain, at least for the current approaches that specifically target the PNS and spinal cord. AAV9 has also been reported to transduce the CNS after intravascular delivery, suggesting the ability of this serotype to cross the blood brain barrier (BBB) [43,44].

AAV2 and AAVrh10 are the only serotypes that have been clinically tested in humans in the CNS. The safety of intraparenchymal infusion of up to 1 × 10^12^ GC of AAV2 to the brain has been demonstrated in the setting of Canavan disease [64], late infantile neuronal ceroid lipofuscinosis, LINCL [40], Parkinson disease [37,41,65-67], and Alzheimer disease [54]. AAVrh10 showed comparable safety for doses up to 7.2 × 10^11^ GC, while achieving higher brain penetration after intraparenchymal infusion compared to AAV2 [33] and was clinically tested for mucopolysaccharidosis IIIA [36] and LINCL [68].

Recent advances in AAV biology showed promise in developing AAV particles with improved transduction efficiency or tissue tropism. The improved transduction efficiency has been demonstrated by using self-complementary (sc-) AAV vectors [69] and while no sc-AAV has so far been used in the CNS in humans, systemic delivery of sc-AAV8 showed safety in the clinical trial for hemophilia B [49]. The improved tissue tropism of AAV has been achieved by rational capsid engineering [70]. While most of these studies are in the early phases of development, one of the novel serotypes termed AAV2.5, selected for muscle tropism, has been brought to a phase I clinical trial for Duchenne muscle dystrophy [71]. IT administration of AAV2.5 in NHP resulted in a transduction pattern similar to that of AAV9 [35], making AAV2.5 an appealing alternative to AAV9 for AAV for pain.

In regards to AAV for pain, we would arrive at different conclusions for the two delivery routes under consideration: IG administration of AAV2 or AAVrh10 as well as IT administration of AAV9 might not need any further investigations of serotype-specific toxicities and might only need to be further assessed in regards to the toxicities of a delivered therapeutic gene (as detailed in Section 5 below). IT AAV8 requires further biodistribution studies to confirm whether its feasible characteristics observed rodents also apply to large animals, which will then determine whether AAV9 or AAV8 would the vector of choice for the IT route in the clinics.

## Potential toxicities associated with the therapeutic genes: Pharmacology

In this section, the review will focus on two transgenes that appear particularly promising for translating AAV gene therapy for pain: prepro-ß-endorphin (ppßE) and interleukin-10 (IL-10). The therapeutic efficacies of these two genes have been investigated and validated in multiple vector systems including AAV by several independent groups including ours [61,72-74]. The ppßE gene was originally developed by us for the use in pain gene therapy [75]. A number of other analgesic genes have been reported in a variety of vector systems and might be alternative candidates for the clinical translation of AAV for pain, whereby the pharmacological assessment will follow similar principles as laid out here for ppßE and IL-10.

### Dose–response relationship and therapeutic index: Transgene as a drug

Effects of the transgene products can be assessed by applying the principles established in pain pharmacology for conventional (non-gene therapy) drugs. Most analgesic drugs, including the peptide ß-endorphin (ßE, considered as a gene-encoded version here), show dose dependence in regards to both their analgesic- and toxic effect. Low doses of analgesic drugs thereby exert low (if any) pharmacological effect and dose escalation results in an analgesic effect and eventually, if doses exceed a certain threshold, the gradual emergence of toxicity. The drug level needed to produce the toxic effect relative to a therapeutic effect is the therapeutic index.

The emphasis on the dose–response relationship and the therapeutic index in pain pharmacology provides important guidance in regards to the development of AAV for pain. A certain degree of toxicity may be expected at high-therapeutic doses and may be not only acceptable but even the goal of dose-escalation in order to optimize the therapeutic benefit. The rationale for delivering the ppßE gene by AAV is the expectation of a favorable therapeutic index. The basis for its safe development will be the appropriate choice of the initial vector dose in a phase I/II trial.

### Tissue-specific transgene expression: improving the therapeutic index

Conventional analgesic drugs, such as opioids, administered orally or intravenously expose all sites of the body to similar drug concentrations. In contrast, AAV gene therapy for pain by the IT or IG route aims at targeting anatomical sites known to mediate analgesic efficacy of opioids but not toxicity: the spinal compartment of the nervous system including the DRGs and the posterior horn of the spinal cord.

IT delivery improves the therapeutic index of conventional opioids. This principle is firmly established through animal studies [11,12] and the use of IT pump-mediated delivery of opioids in patients with the most severe pain states [76]. Vector systems that were initially used in gene therapy for pain, such as adenovirus [77,78] or plasmids [74,79] transduced only meningeal lining fibroblasts, which secreted the transgene product into the spinal CSF emulating an IT pump.

An important observation in the field of AAV for pain, however, has been the marked tropism of AAV for DRG neurons if administered IT or IG. This was a very favorable, while unexpected, finding and has subsequently been confirmed by several groups in different animal models [16,18,59-61,80-82]. It has an important promising implication for the pharmacology of AAV for pain: the potential to not only match the therapeutic index of IT opioids but to improve further upon it. AAV-mediated transgene pharmacokinetics can be formalized as follows. If the transgene encodes a secreted protein such as ppßE and IL-10, the protein can reach the target cells by two routes: (i) through release into the CSF, i.e., emulating an IT pump, and (ii) in an autocrine/paracrine fashion within the immediate surrounding of the transduced cells. While the ratio of biological effects (ii)/(i) cannot be directly measured in animal models, it may be an important consideration when assessing the viability of AAV gene therapy for pain explicating why the therapeutic index of AAV will likely be improved not only over systemic opioids but also when compared with IT pump delivery of opioids.

Of note, a high degree of DRG-neuron selectivity has been the fundamental rationale for the HSV-based approach to pain gene therapy by the subcutaneous route of vector administration [4,83]. IT AAV may be less selective for DRG neurons than subcutaneous HSV, but will likely improve the therapeutic index over IT delivery of conventional opioids (as discussed above). Furthermore, the IG route will very likely yield the most DRG neuron-selective transgene activity achievable with AAV potentially matching the DRG neuron-selectivity of subcutaneous HSV.

### Specific consideration for ppßE

ßE, the secreted peptide product of ppßE, is a μ-opioid receptor agonist and thereby shares its pharmacological activity with the most commonly used conventional opioids such as morphine. The toxicological assessment of ppßE can therefore be guided by the robust clinical experience with exogenous opioids. In addition, ßE was tested in clinical trials.

The principal sites where opioid toxicity occurs at high doses are the CNS and the periphery. The most common adverse effects of opioids in the CNS are nausea and vomiting, mental status changes, pruritus, urinary retention, and respiratory depression [84-87], while the peripheral adverse effects include constipation, cardiovascular adverse events, derangement of hypothalamo-pituitary axis, and immune-modulation [87-91]. Both the CNS and peripheral adverse effects are dependent on the drug concentration at a given site and may be reversed by naloxone, a μ-, κ-, and δ-opioid receptor antagonist.

When IT administration of ß-endorphin was tested in humans, it resulted in profound analgesia similar to that attainable with exogenous opioids [92-97]. Dose escalation led to a toxicity profile that would be expected with an IT opioid drug, whereby doses of IT ßE of up to 7.5 mg resulted in miosis, drowsiness, and elevation plasma prolactin levels in some patients. No systemic adverse effects related to the treatment were found in those studies, reflecting the inability of ß-endorphin to cross the BBB, keeping it from escaping from the IT space to the systemic circulation.

In conclusion, the therapeutic and toxic effects of ppßE are expected to be vector dose-related and the specific toxicities can be predicted from the previous clinical experience with IT opioids and ßE. What remains to be defined in the preclinical large animals studies is the maximum tolerated dose (MTD) of the vector encoding ppßE for both the IT and the IG route. A phase I/II clinical would then test safety for vector doses starting with the lowest potentially efficacious dose and sequentially being escalated towards the MTD established in animals.

### Specific consideration for IL-10

IL-10 is of interest for pain therapy because it may be the first/only available agent to target a mechanism of chronic pain first described about a decade ago, glial activation [98]. Recombinant IL-10 administered IT has been found to be efficacious in animal models of chronic pain [99,100]. The use of recombinant protein, however, has not been tested in humans due to its short half-life of IL-10 in the CSF, its inability to cross the BBB, and the impracticality of continuous IT delivery over a prolonged period of time [78]. A pharmaceutical formulation of IL-10 needed for delivery by an IT pump, i.e. the stability at ambient temperature and the high concentration, has proved to be difficult. Therefore, gene therapy may provide the best (or only) strategy for testing targeted spinal delivery of IL-10 for pain in the clinical setting.

While toxicity of IL-10 delivery targeted to the PNS by either the IT or the IG route has not been addressed in large animal models, rodent data suggest good tolerability of IL-10 in the CSF [61,74,101,102]. Furthermore, systemic toxicities of IL-10 have been thoroughly characterized in trials of recombinant IL-10 for Crohn disease, rheumatoid arthritis, Wegener granulomatosis, and psoriatic arthritis [103-107]. Safety studies showed that a single intravenous injection of up 100 μg/kg IL-10 in healthy humans led to transient, dose-related flu-like symptoms accompanied by neutrophilia, monocytosis, lymphopenia, and down-regulation of pro-inflammatory cytokines IL-1 and TNF-α [108]. Long-term intravenous or subcutaneous administration of IL-10 in rats and NHP confirmed hematological effects of IL-10 and also showed its inhibitory effect on T-lymphocytes [109].

Establishing the MTD for AAV encoding IL-10 will be an important goal for both the IT and the IG route. Clinical testing would then start at a lower vector dose with subsequent dose escalation to establish the MTD in humans.

### Transgene-specific immune response

Syngeneic transgenes will not induce serological or cellular immunity. Therefore, no immunity is to be expected in a clinical trial using human IL-10 as therapeutic gene and no special studies appear to be necessary towards assessing it.

The case of ppβE may require additional consideration, because the gene includes an artificial fusion site. Specifically, the pre-pro sequence of ppβE (the amino-terminal “pp” part of the protein) is taken from nerve growth factor (NGF); thus, this portion of the gene by itself is not of concern because it can be made syngeneic (by taking the pre-pro sequence from human NGF). The carboxy terminal portion of ppβE gene encodes the pharmacologically active βE peptide. By itself this portion of the gene is not of concern either, because it can also be made syngeneic (by using the βE sequence as it occurs in the precursor protein human pro-opiomelanocortin (POMC)). In the ppβE gene, these two syngeneic peptides are fused creating a potentially non-syngeneic epitope.

The rationale for using ppβE rather than POMC is to circumvent the requirement of POMC to be processed in acidified secretory vesicles found only in certain cell types and its reliance on the regulated secretory pathway. ppβE can be processed into βE by all cell types and secretion of βE occurs *via* the constitutive secretory pathway yielding continuous release of the pharmacologically active βE peptide, which is the desired property of this therapeutic gene. Because the processing of the ppβE peptide into its syngeneic components, the pre-pro-sequence and βE, occurs immediately following its ribosomal synthesis and co-translational transport into the ER, the fusion epitope may not even have a chance to become immunologically apparent. Therefore, to evaluate the possibility of cellular immune toxicity, pre-clinical toxicity studies of ppβE will have to assess the integrity of transduced tissues such as neuropathological evaluation of DRG.

A challenge for pre-clinical toxicology assessments is whether to perform animal testing with syngeneic transgenes, i.e., specifically adapted to the animal species employed, or to perform testing of the human-type vector slated to be used in a clinical trial, i.e. xenogeneic to the animal model. Xenogeneic transgenes are well-tolerated in rodents, which express even the bacterial EGFP gene long term. However, xenogeneic transgenes induced immunity in large animals [32]. As a consequence, an AAV vector encoding a species-specific, i.e., syngeneic transgene could be used for preclinical toxicology studies of AAV for pain. The vector that is ultimately used in the clinic could also be given to animals, but the latter type of studies should be limited to effects observable within approximately one week because all later effects can be expected to be confounded by a xenogeneic immune response that is an artifact of the testing scenario and not applicable to the anticipated scenario of a clinical trial.

## Conclusions

Responsible and effective preclinical-to-clinical translation of AAV for pain represents a substantial scientific and regulatory challenge. Yet, the goal appears to be supported by two considerations: The growing clinical research experience with AAV (for non-pain applications) and the need for innovative clinical research in patients with intractable pain from incurable cancer. The following two sections follow these two notions in an attempt to turn our review findings into a proposed action plan.

### Preclinical toxicity assessment

#### Additional studies required

For the IT route of AAV delivery, preclinical animals studies are needed to

● Establish the MTD of AAV vectors

◦ Assess health of animals clinically

◦ Neuropathological assessment of neural structures such as the DRG

◦ Repeat independent experiments for each specific vector design: transgene, serotype

◦ If MTD cannot be reached, document absence of toxicity at a dose exceeding the highest concentration anticipated in humans, e.g., if dose range contemplated in humans is 10^11^ to 10^13^ GC, then test up to 10^14^ GC in a large animal

● Establish biodistribution

We propose that testing in large animals would be more meaningful than testing in rodents, even if performed in a significantly smaller number of animals, because a species such as the pig better resembles the mechanical-anatomical and immunological characteristics of humans than rats. We propose that testing for the first transgene-capsid combination should be done in two iterations: a long-term experiment (such as 3 months follow up) with the syngeneic transgene (such as made for the pig) and a short-term experiment (such as ten days) with the human transgene (that would be xenogeneic in pigs). For subsequent assessments of other transgene-vector combinations, it may be justified to limit studies to the vector that is to be used in the clinical trials expressing the human transgene version.

For the IG route of AAV delivery, additional preclinical animal studies are needed to

● Confirm the safety of placing a CED needle into the DRG for vector infusion

#### Existing studies to cross-reference

Existing studies reviewed above have already addressed the following issues:

● General vector related toxicities including insertional mutagenesis, germ line transmission, replication escape, and dissemination of the vector to the public domain—AAV vector administration *per se* appears non-hazardous

● Serologic response against AAV (*sin* transgene)

#### Issues outside the realms of animal models

Modeling human immunity to vectors in animal models has remained challenging and uncertain. While numerous experimental approaches and assays have been used or proposed, predictions from rodents appear to be misleading if applied to humans and predictions from various large animal models uncertain at best. The conservative assumption therefore has to be that immune toxicity is an anticipated untoward effect requiring close monitoring in the clinical setting to protect patients and learn more about AAV vector biology in the clinical setting.

### Hypothetical design of a phase I/II clinical trial

Most gene therapy trials for non-malignant disorders have been conducted in patients with a near-normal life expectancy, such as in the case of hemophilia B. The clinical trial of AAV for pain that we envision would substantially differ in terms of target population: Patients with an unacceptably high symptom burden from intractable pain at the end of life in the setting of a progressing cancer that is incurable or even untreatable (by antineoplastic therapies). Enrollment requirements could include a minimum- and a maximum life expectancy. Both could be shorter for early patient cohorts to limit the impact that unexpected adverse effects could have, such as a range of 4 to 8 weeks. As safety is established throughout the course of the trial, the minimum life expectancy could become longer, e.g., 3 months, to allow collection of data on longer-term effects. While the same high scientific standards should apply to a palliative-care clinical trial in cancer patients as to trials in patients with non-malignant diseases, the proposed clinical trial setting may be the most appropriate for a pre-clinical to clinical research transition in a case such as AAV gene therapy, where toxicities cannot be modeled perfectly in animals.

A phase I/II clinical trial would likely focus first on the transgene with the best-known pharmacology, i.e., an opioid. ppßE may therefore be the most suitable candidate for early clinical translation thanks to the extensive clinical experience with exogenous IT opioids. In addition, the spread of the vector suspension in the IT space for a given volume can be guided by the experience with IT drugs such as methotrexate, which distribute within the CSF-filled space and are routine given as in a volume of 5 cc and may be safe for injection volumes of up to 10 ml in adults.

An exciting medium- to long-term impact of studying AAV for pain in the cancer center setting may be the opportunity to gain insights into AAV biology in humans. Given that some patients may consent to postmortem tissue collection (an autopsy) a trial in this setting may offer a unique chance for analyzing tissue samples from human subjects receiving AAV at a markedly more rapid turnaround than in previous AAV clinical trials.

Another long-term potential from the perspective of analgesic treatment development is that AAV gene therapy may serve as a platform for investigating novel analgesic targets. For instance, the efficacy of IL-10 would test the analgesic effects of counter-acting glial activation in the setting of the human pain state, which could then guide development of conventional (small molecule) drugs harnessing the same mechanism of action. While IL-10 has been proposed as an analgesic strategy more than a decade ago, clinical testing could not be performed to date presumably because prolonged IT delivery of recombinant IL-10 is impractical. AAV could be the means to bring novel analgesic strategies such as IL-10 from the laboratory into clinical trials.

## Competing interests

The authors declare that they have no competing interests. RJS is a founder of Asklepios Biopharmaceutical, Inc.

## Authors’ contributions

JP and ASB wrote the manuscript. LFH prepared the figure. CHE, AA, and RJS contributed sections on adeno-associated viral vectors. All authors read and approved the final manuscript.
